# Anatomical Variability of Mandibular Root Canal Systems in a Saudi Clinical Sample: A CBCT Study Using Ahmed and Vertucci Classifications

**DOI:** 10.1155/ijod/8277155

**Published:** 2026-10-29

**Authors:** Ahmed A. Almalaq, Dalal H. Alshammary, Khaled M. Bendary, Firas J. Shaini, Ahmed Alrusyni, Wesam F. Mohamed, Reem A. Alkhamsan, Mahdi M. Al Wusaybie, Khalid N. Alshammari, Turki M. Alazmi, Tahani M. Alharbi, Salem A. Alhamandi, Faisal A. Alrashid, Ahmed A. Madfa

**Affiliations:** ^1^ Endodontics Department Hail Dental Center Hail Health Cluster Ministry of Health Hail Saudi Arabia moh.gov.sa; ^2^ Department of Restorative Dental Science College of Dentistry University of Ha’il Ha’il Saudi Arabia uoh.edu.sa

**Keywords:** Ahmed classification, C-shaped canals, cone beam computed tomography, mandibular teeth, root canal morphology, Saudi population

## Abstract

**Background:**

Understanding anatomical variations in root and canal morphology is essential for successful endodontic treatment. Studies based on clinical populations provide valuable data for clinicians treating patients with similar demographic and anatomical characteristics. This study evaluated root and canal morphology of mandibular teeth in a Saudi clinical sample using cone beam computed tomography (CBCT).

**Methods:**

This retrospective cross‐sectional study analyzed CBCT scans of 150 patients from Hail Dental Center, Saudi Arabia. A total of 1104 mandibular teeth (with the individual tooth serving as the unit of analysis) were evaluated using a Carestream CS 8100 3D system (0.15 mm voxel size). Three calibrated endodontists independently assessed root number, canal configurations using Vertucci and Ahmed classification systems, and C‐shaped canals using Fan’s classification.

**Results:**

Single‐rooted teeth comprised 83.2% (*n* = 918) of the sample, while two‐rooted teeth accounted for 16.6% (*n* = 183). The Ahmed–Dummer classification revealed single canal configuration (^1^) in 61.2% (*n* = 676), followed by ^121^ (13.0%), M^1^D^1^ (8.2%), and ^12^ (7.9%). C‐shaped canals were identified in 2.2% (*n* = 24) of teeth, with highest prevalence in first premolars (7.2%), second molars (6.4%), and second premolars (3.3%).

**Conclusions:**

This study provides comprehensive data on mandibular tooth morphology in a Saudi clinical sample. Significant anatomical variations, particularly in premolars and molars, emphasize the importance of preoperative CBCT assessment. The Ahmed–Dummer classification proved valuable for documenting complex anatomical variations beyond traditional classification systems.

## 1. Introduction

A thorough understanding of root canal anatomy is essential to the biological and technical effectiveness of endodontic therapy [1, 2]. Clinical factors, including preoperative periapical status, root filling quality, and coronal restoration, have been associated with primary root canal treatment outcomes [3]. One of the primary causes of chronic infection and post‐treatment illness is still the failure to identify and treat the entire root canal system [4, 5]. Effective cleaning, shaping, and obturation may be hampered by anatomical differences, such as extra roots, supplementary canals, intricate isthmuses, and unusual canal layouts [1, 4]. Several studies have shown that root and canal morphology differ significantly between various populations, ethnic groupings, and geographical areas [6–9]. For example, there is significant variation among races in the occurrence of three‐rooted mandibular molars [7, 8], and Asian groups are more likely than Caucasian populations to have C‐shaped canal configurations [8, 9]. These variations demonstrate how crucial population‐specific anatomical information is for directing clinical practice and enhancing treatment predictability. However, there is still a dearth of information on the morphology of mandibular teeth in Saudi populations [7, 9].

The study of root canal anatomy has greatly benefited from cone beam computed tomography (CBCT), which allows for high‐resolution, non‐destructive, three‐dimensional assessment [10–12]. High‐resolution micro‐computed tomography has also demonstrated the complexity of internal root canal geometry and enabled detailed three‐dimensional characterization of canal anatomy [13]. CBCT allows for precise in vivo evaluation of root number, canal layout, and complicated internal morphology without causing structural damage, in contrast to traditional radiography or tooth‐clearing procedures [10, 12]. It is especially useful for diagnosing complex anatomical characteristics including isthmuses, auxiliary canals, and C‐shaped canal networks [10, 12].

The most popular approach for classifying canal topologies is still the Vertucci classification system [1], despite its acknowledged shortcomings in explaining the intricate multirooted anatomy and independent root variants. In order to overcome these limitations, the Ahmed classification system [6] was developed. It uses an alphanumeric coding system that enables the independent identification of each root and its canal layout using specific root designations (e.g., mesial [M] and distal [D]). This method offers a more thorough and useful description of complicated morphologies for therapeutic settings.

C‐shaped canal configurations deserve special investigation because of their clinical relevance and well‐documented diversity in incidence among populations [9, 14, 15]. Their intricate structure makes debridement difficult, raises the possibility of procedural mistakes, and, if neglected, might lead to partial obturation [9, 14]. A significant number of thorough CBCT‐based studies assessing mandibular tooth morphology in Saudi populations using both the Vertucci and Ahmed categorization systems simultaneously are still lacking, despite growing global evidence [7, 16]. Additionally, information about the distribution and frequency of C‐shaped canals in all mandibular tooth types within this group is still incomplete and inadequately investigated [7, 9, 15].

The purpose of this study was to use CBCT imaging to thoroughly assess the root and canal morphology of mandibular teeth in a Saudi clinical sample. This included determining the number of roots and canal configurations based on the Vertucci and Ahmed classification systems as well as determining the frequency and distribution of C‐shaped canals in all mandibular tooth types.

## 2. Methods

### 2.1. Study Design

This retrospective cross‐sectional observational study evaluated the root and root canal morphology of mandibular teeth using CBCT. Ethical approval was obtained from the Hail Dental Centre, Hail Health Cluster, Ministry of Health, Saudi Arabia (Approval Number 2019‐26). The requirement for informed consent was waived because this retrospective study involved the analysis of previously acquired and anonymized CBCT records. Prior to the analysis, all datasets were anonymized in compliance with institutional rules to guarantee data security and confidentiality. This study was conducted and reported in accordance with the Strengthening the Reporting of Observational Studies in Epidemiology (STROBE) guidelines.

### 2.2. Sample Size Calculation

A single‐proportion calculation based on the expected frequency of C‐shaped canals in mandibular teeth was used to determine the sample size. The formula used was *n* = [*Z* ^2^ × *P*(1−*P*)] / *d* ^2^, where *Z* = 1.96 (for 95% confidence level), *p*  = expected prevalence (0.20), and *d* = margin of error (0.05). The minimum necessary sample size was determined to be 246 teeth, assuming a 95% confidence level, a 5% margin of error, and an expected prevalence of 20% based on prior regional studies. A significantly larger sample was added to improve statistical power, allow subgroup analysis by sex and tooth type, and account for probable exclusions. In the end, 1104 mandibular teeth from 150 patients satisfied the eligibility requirements and were examined, surpassing the minimal sample size needed and enhancing the accuracy of prevalence estimations. The unit of analysis was the individual tooth, not the patient. It should be noted that this sample size calculation was based exclusively on the prevalence of C‐shaped canals as the primary outcome, and the study was not specifically powered for every secondary analysis (e.g., root number, canal configurations, and gender comparisons).

### 2.3. Sample Selection

CBCT scans acquired at the Hail Dental Center between January 2020 and December 2022 underwent eligibility screening. Because CBCT examinations were performed for specific clinical indications, the study population represents a selected clinical sample of patients attending this tertiary referral dental center rather than the general Saudi population.

#### 2.3.1. Inclusion Criteria

High‐quality CBCT scans with sufficient image resolution were included. Eligible teeth demonstrated fully developed roots with complete apical formation and belonged to patients aged ≥ 18 years. In addition, included teeth had no history of previous root canal treatment, post placement, or full‐coverage crown restoration and showed no evidence of root resorption, canal calcification obstructing canal morphology, or periapical pathology.

#### 2.3.2. Exclusion Criteria

CBCT scans with inadequate image quality or insufficient resolution were excluded. Teeth with incomplete root development, previous endodontic treatment, posts, or full‐coverage crowns were also excluded. Additional exclusion criteria included the presence of significant pathological changes affecting root canal morphology, such as root resorption, obstructive calcifications, or periapical lesions, as well as mandibular third molars.

### 2.4. CBCT Imaging Protocol

A Carestream CS 8100 3D machine (Carestream Dental LLC, Atlanta, GA, USA) was used to capture all scans using the following standard parameters: 90 kVp, 4 mA, 0.15 mm voxel size, 8 cm × 8 cm field of view, and 14 s exposure duration. CS 3D Imaging Software (Carestream Dental) was used to rebuild and evaluate the images utilizing multiplanar reconstruction in axial, sagittal, and coronal perspectives.

### 2.5. Image Analysis and Examiner Calibration

The scans were independently assessed by three experienced endodontists, each with more than 5 years of clinical experience. Twenty randomly chosen CBCT scans (about 140 teeth) that were not part of the research sample were used for calibration prior to official evaluation. This sample was considered sufficient, as it encompassed a representative variety of tooth types (incisors, canines, premolars, and molars) and included diverse anatomical complexities to establish uniform interpretation standards. The calibration scans were randomly selected from the institutional archive, ensuring they met the same general inclusion criteria (high‐quality, fully developed roots), but were excluded from the main study sample to avoid bias. Examiners evaluated these scans on their own, and then, they reached an agreement to establish uniform interpretation standards. Any disagreements among the three independent examiners during the main study evaluation were resolved through joint discussion and consensus. In cases where consensus could not be reached, a senior endodontist with more than 10 years of experience was consulted to make the final decision.

Fleiss’ kappa coefficient was used to determine interobserver reliability, and the results showed considerable to excellent agreement (*κ* = 0.82 for root number, *κ* = 0.79 for canal configuration, and *κ* = 0.84 for C‐shaped canal identification). After 2 weeks, 150 randomly chosen teeth (13.6% of the sample) were reassessed to determine intraobserver reliability. The results showed high agreement (*κ* = 0.86–0.91 for the root number, *κ* = 0.83–0.89 for the canal structure, and *κ* = 0.88–0.93 for C‐shaped canals).

The number of roots, the type of root (separate or fused), the number of canals at the coronal, middle, and apical thirds, the canal configuration based on the Vertucci and Ahmed classification systems, and the presence and arrangement of C‐shaped canals based on Fan’s classification were all noted for every tooth.

### 2.6. Statistical Analysis

Data were analyzed using descriptive and inferential statistics. Categorical variables were expressed as frequencies and percentages. The distribution of root and canal morphology was evaluated according to tooth type and sex using the chi‐square test. Inter‐ and intraexaminer reliability were assessed using Fleiss’ kappa coefficient, with *κ* > 0.75 indicating substantial agreement. Statistical significance was set at *p*  < 0.05.

## 3. Results

### 3.1. Sample Characteristics

A total of 150 patients (78 males and 72 females; mean age 34.6 ± 12.3 years, range 18–65 years) contributed 1104 mandibular teeth to this study (Table 1). The sample comprised 528 teeth from male patients (47.8%) and 576 teeth from female patients (52.2%). Tooth type distribution was as follows: central incisors (*n* = 200, 18.1%), lateral incisors (*n* = 201, 18.2%), canines (*n* = 196, 17.8%), first premolars (*n* = 181, 16.4%), second premolars (*n* = 150, 13.6%), first molars (*n* = 82, 7.4%), and second molars (*n* = 94, 8.5%). The study comprehensively illustrates multiple significant anatomical variations in the mandibular teeth, as demonstrated across the radiographic findings shown in Figures 1–7. These variations are detailed below according to root morphology, canal configuration classification systems, and C‐shaped canal characteristics.

**Figure 1:**
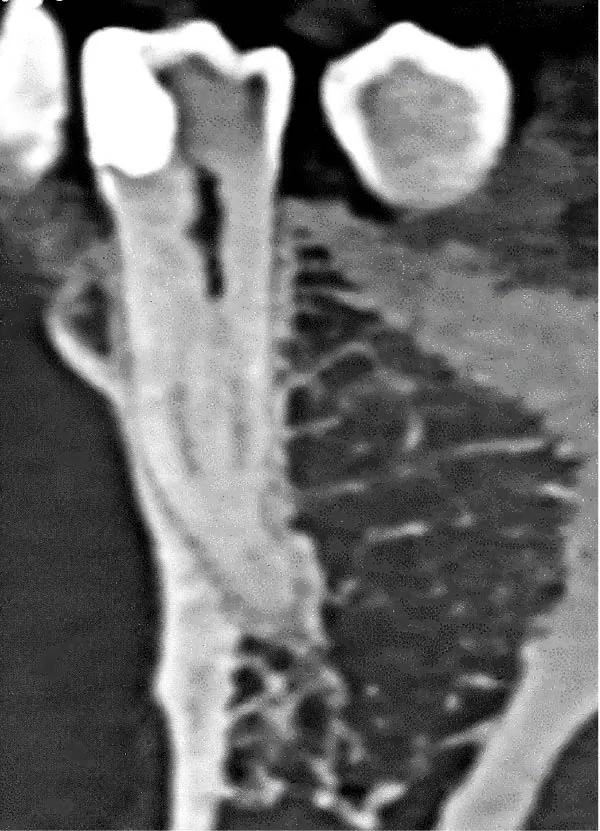
Coronal view of a mandibular premolar showing two canals merging apically (Vertucci type II configuration).

**Figure 2:**
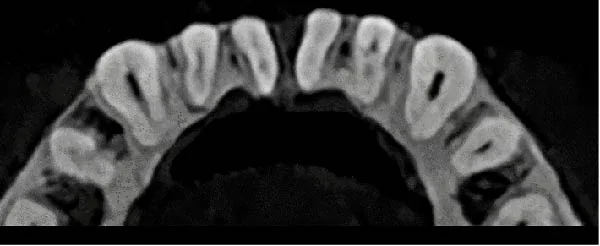
CBCT axial view of mandibular anterior teeth demonstrating additional (lingual) root canals.

**Figure 3:**
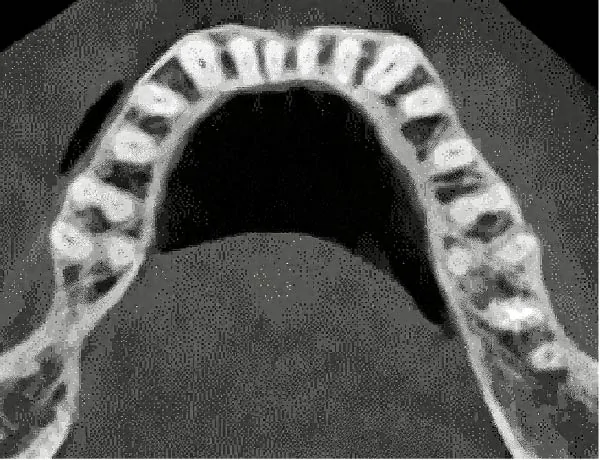
Axial CBCT view of the mandible showing bilateral radix entomolaris in mandibular first molars.

**Figure 4:**
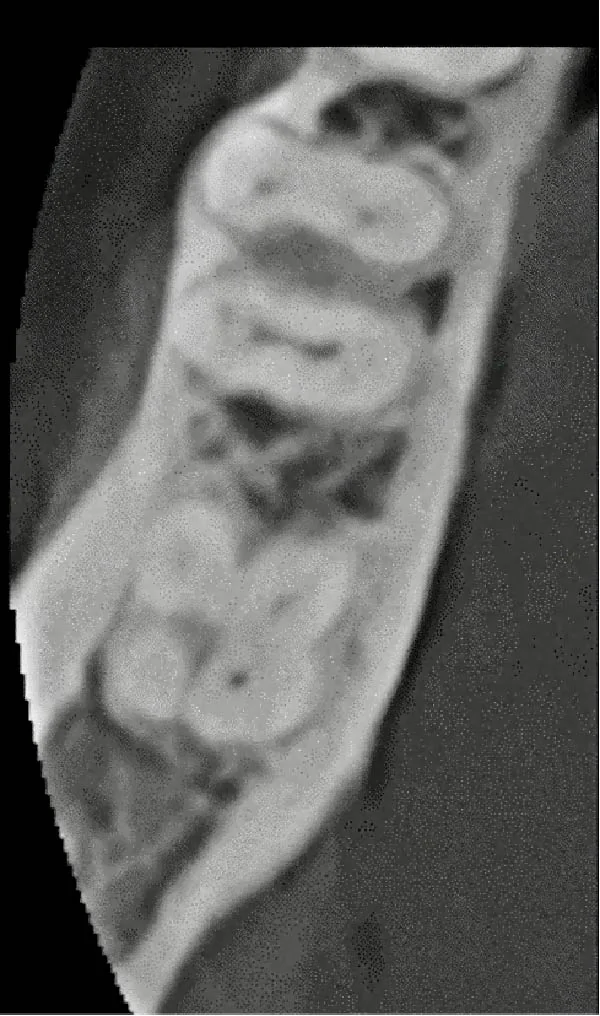
Axial CBCT image of a mandibular right second molar (tooth 47) exhibiting a rare four‐rooted configuration.

**Figure 5:**
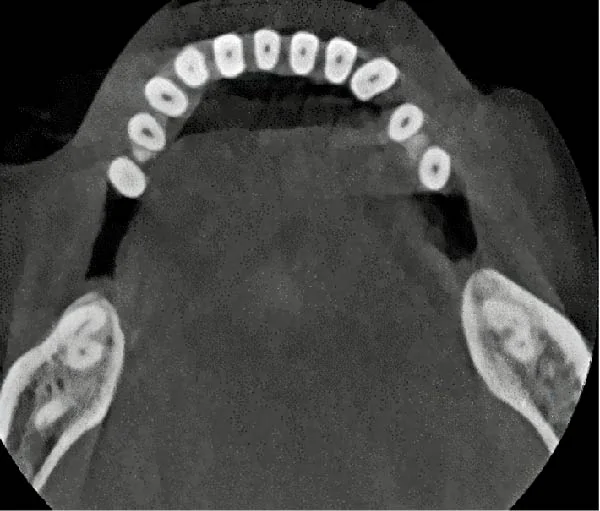
CBCT image confirming bilateral C‐shaped canal morphology in mandibular second molars.

**Figure 6:**
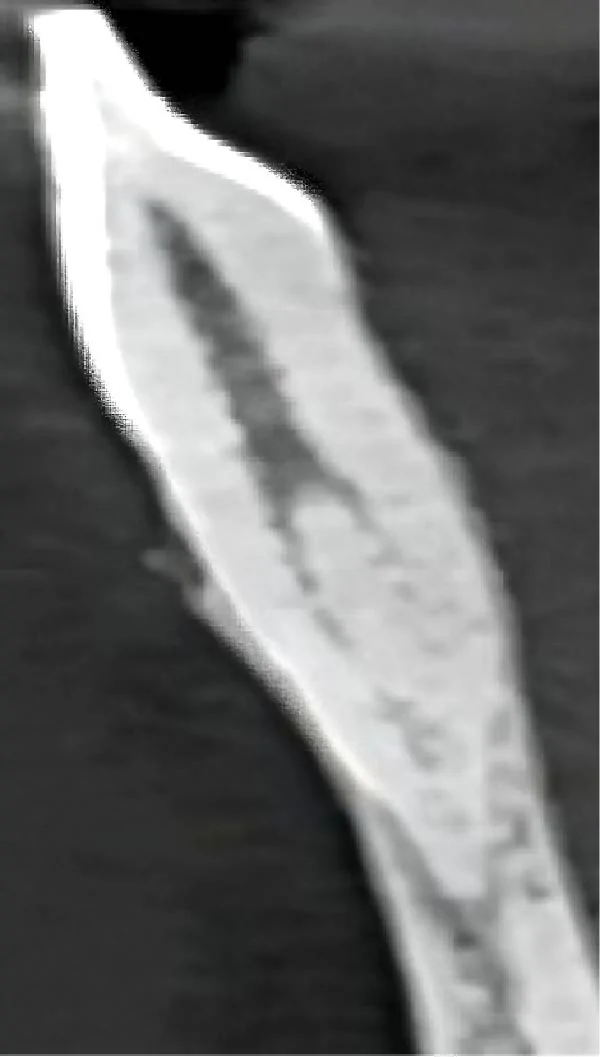
Sagittal/oblique view of a mandibular canine with a Vertucci type III canal system.

**Figure 7:**
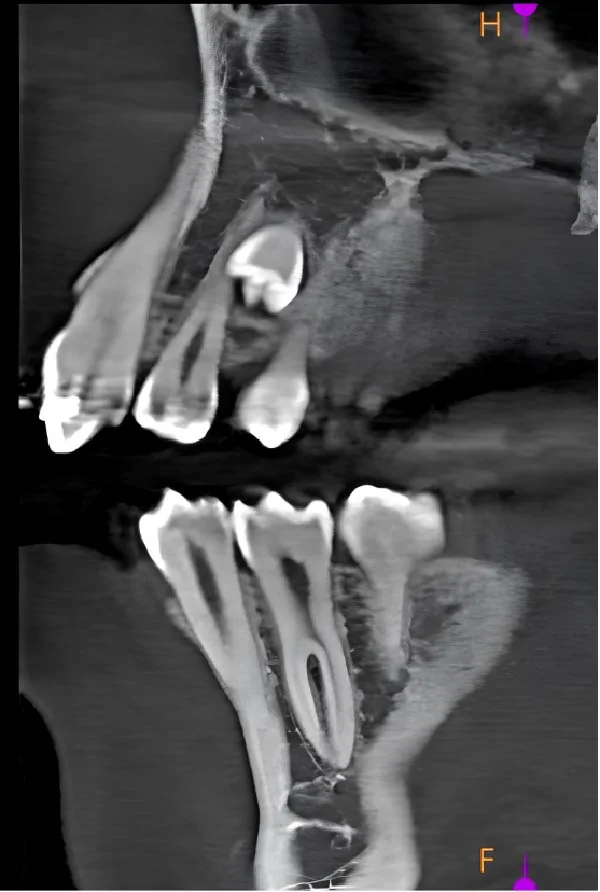
Mandibular premolar displaying two completely separate roots.

**Table 1:** Distribution of root number and fused roots by tooth type (*n* = 1104).

Tooth type	Total (*n*)	Single root, *n* (%)	Two roots, *n* (%)	Three roots, *n* (%)	Four roots, *n* (%)	Fused roots, *n* (%)^a^
Central incisor	200	200 (100.0)	0 (0.0)	0 (0.0)	0 (0.0)	0 (0.0)
Lateral incisor	201	201 (100.0)	0 (0.0)	0 (0.0)	0 (0.0)	0 (0.0)
Canine	196	195 (99.5)	1 (0.5)	0 (0.0)	0 (0.0)	0 (0.0)
First premolar	181	174 (96.1)	7 (3.9)	0 (0.0)	0 (0.0)	7 (3.9)
Second premolar	150	146 (97.3)	4 (2.7)	0 (0.0)	0 (0.0)	2 (1.3)
First molar	82	1 (1.2)	80 (97.6)	1 (1.2)	0 (0.0)	0 (0.0)
Second molar	94	1 (1.1)	91 (96.8)	1 (1.1)	1 (1.1)	7 (7.4)
Total	**1104**	**918 (83.2)**	**183 (16.6)**	**2 (0.2)**	**1 (0.1)**	**16 (1.5)**

*Note:* Bold values represent the overall totals for the study sample and are used to distinguish the total row without indicating statistical significance.

^a^Fused roots: roots joined together for part or all of their length. Percentages calculated from total teeth per tooth type.

### 3.2. Root Number and Type Distribution

Analysis of root number revealed that single‐rooted teeth comprised 83.2% (*n* = 918) of the total sample (Table 2), while two‐rooted teeth accounted for 16.6% (*n* = 183). Three‐rooted teeth were rare (*n* = 2, 0.2%), and only one four‐rooted tooth (0.1%) was identified. All anterior teeth (central incisors, lateral incisors, and canines) exhibited a single‐rooted morphology, with the exception of one two‐rooted canine (0.5%) (Figure 2). Among premolars, first premolars demonstrated 96.1% (*n* = 174) single‐rooted and 3.9% (*n* = 7) two‐rooted morphology, while second premolars showed 97.3% (*n* = 146) single‐rooted and 2.7% (*n* = 4) two‐rooted patterns. First molars (*n* = 82) exhibited a predominantly two‐rooted morphology (97.6%, *n* = 80), with 1.2% (*n* = 1) single‐rooted and 1.2% (*n* = 1) three‐rooted specimens (Figure 3). Second molars displayed 1.1% (*n* = 1) single‐rooted, 96.8% (*n* = 91) two‐rooted, 1.1% (*n* = 1) three‐rooted, and 1.1% (*n* = 1) four‐rooted configurations (Figure 4).

**Table 2:** Root canal configuration by tooth type according to Vertucci classification (*n* = 1104).

Tooth type	Total (*n*)	Type I, *n* (%)	Type II, *n* (%)	Type III, *n* (%)	Type IV, *n* (%)	Type V, *n* (%)	Type VI, *n* (%)	Type VII, *n* (%)	Type VIII, *n* (%)	Other^a^, *n* (%)
Central incisor	200	176 (88.0)	0 (0.0)	13 (6.5)	0 (0.0)	11 (5.5)	0 (0.0)	0 (0.0)	0 (0.0)	0 (0.0)
Lateral incisor	201	180 (89.6)	0 (0.0)	11 (5.5)	0 (0.0)	10 (5.0)	0 (0.0)	0 (0.0)	0 (0.0)	0 (0.0)
Canine	196	185 (94.4)	0 (0.0)	7 (3.6)	0 (0.0)	4 (2.0)	0 (0.0)	0 (0.0)	0 (0.0)	0 (0.0)
First premolar	181	87 (48.1)	0 (0.0)	13 (7.2)	7 (3.9)	61 (33.7)	0 (0.0)	0 (0.0)	0 (0.0)	13 (7.2)
Second premolar	150	121 (80.7)	0 (0.0)	8 (5.3)	4 (2.7)	12 (8.0)	0 (0.0)	0 (0.0)	0 (0.0)	5 (3.3)
First molar	82	1 (1.2)	0 (0.0)	0 (0.0)	0 (0.0)	0 (0.0)	0 (0.0)	0 (0.0)	0 (0.0)	81 (98.8)
Second molar	94	1 (1.1)	0 (0.0)	0 (0.0)	0 (0.0)	0 (0.0)	0 (0.0)	0 (0.0)	0 (0.0)	93 (98.9)
Total	**1104**	**751 (68.0)**	**0 (0.0)**	**52 (4.7)**	**11 (1.0)**	**98 (8.9)**	**0 (0.0)**	**0 (0.0)**	**0 (0.0)**	**192 (17.4)**

*Note:* Type I = single canal; type II = two canals joining near apex; type III = one canal dividing then rejoining; type IV = two separate canals; type V = one canal dividing into two; type VI = two canals joining, then dividing; type VII = one canal dividing, rejoining, then dividing again; type VIII = three separate canals. Bold values indicate the overall totals and corresponding percentages for each classification category, highlighted for clarity and not representing statistical significance.

^a^Other: multirooted teeth requiring Ahmed–Dummer classification for accurate description (see Table 3).

**Table 3:** Frequency and clinical significance of root canal configurations using Ahmed classification (*n* = 1104).

Rank	Configuration^a^	Frequency, *n* (% of total sample)	Primary tooth types (% within tooth type)^b^	Clinical significance
1	^1^	676 (61.2)	All anterior teeth, premolars	Simple, predictable anatomy; standard instrumentation
2	^121^	143 (13.0)	CI: 65/200 (32.5%), LI: 53/201 (26.4%), canines: 18/196 (9.2%)	Temporary bifurcation; challenging to instrument
3	M^1^D^1^	91 (8.2)	1st molars: 32/82 (39.0%), 2nd molars: 59/94 (62.8%)	Single canal in each root; simpler molar anatomy
4	^12^	87 (7.9)	1st premolars: 31/181 (17.1%), 2nd premolars: 5/150 (3.3%)	Bifurcation requires identification of both canals
5	M^2^D^1^	43 (3.9)	1st molars: 19/82 (23.2%), 2nd molars: 24/94 (25.5%)	Classic molar anatomy; mesial complexity requires careful negotiation
6	M^2^D^121^	16 (1.4)	1st molars: 13/82 (15.9%), 2nd molars: 3/94 (3.2%)	Mesial two canals, distal temporary bifurcation; high complexity
7	M^1^D^12^	8 (0.7)	1st molars: 3/82 (3.7%), 2nd molars: 5/94 (5.3%)	Distal bifurcation adds complexity
8	^2^	7 (0.6)	1st premolars: 2/181 (1.1%), 2nd premolars: 5/150 (3.3%)	Two separate canals throughout; requires dual instrumentation
9	M^2^D^21^	5 (0.5)	1st molars: 5/82 (6.1%)	Distal canal merging; enhanced irrigation needed
10	M^21^D^21^	5 (0.5)	1st molars: 4/82 (4.9%), 2nd molars: 1/94 (1.1%)	Both roots with merging canals; rare and complex
11	^21^	5 (0.5)	Canines: 2/196 (1.0%), premolars: 3/331 (0.9%)	Two canals merging to one; requires careful negotiation
12	M^2^D^2^	4 (0.4)	1st molars: 4/82 (4.9%)	Two canals in each root; increased complexity
13	^131^	2 (0.2)	CI: 2/200 (1.0%)	One canal dividing into three then rejoining; rare configuration
14	M^21^D^121^	2 (0.2)	1st molars: 1/82 (1.2%), 2nd molars: 1/94 (1.1%)	Mesial merging, distal bifurcation; complex anatomy
15	M^12^D^1^	2 (0.2)	2nd molars: 2/94 (2.1%)	Mesial bifurcation, distal single canal
16	Other variants	8 (0.7)	Premolars, molars (includes M^2^D^12^, M^1^D^1a^, M^21^, rare 3/4‐root variants)	Unusual configurations requiring individualized approach

*Note:* ^1^ = single canal throughout; ^12^ = one canal dividing into two; ^121^ = one canal dividing then rejoining; ^2^ = two separate canals; ^21^ = two canals merging to one; ^131^ = one canal dividing into three then rejoining; M = mesial root; D = distal root. Example: M^2^D^1^ = mesial root with two canals, distal root with one canal.

Abbreviations: CI, central incisor; LI, lateral incisor.

^a^Ahmed notation: superscript numbers indicate canal count at different root levels from coronal to apical.

^b^Percentages are calculated using the total number of teeth in the corresponding tooth‐type category as the denominator.

Root fusion was observed in 1.5% (*n* = 16) of the total sample (Table 3). The distribution of fused roots by tooth type was as follows: first premolars 3.9% (*n* = 7), second premolars 1.3% (*n* = 2), and second molars 7.4% (*n* = 7). No fused roots were identified in the anterior teeth or first molars. The remaining 1088 teeth (98.5%) exhibited a separate root morphology.

### 3.3. Vertucci Classification of Canal Configurations

According to the Vertucci classification, type I configuration (single canal from orifice to apex) was most prevalent overall (Figure 1), accounting for 68.0% (*n* = 751) of all teeth examined (Table 4). Anterior teeth predominantly exhibited a Type I morphology: central incisors 88.0% (*n* = 176), lateral incisors 89.6% (*n* = 180), and canines 94.4% (*n* = 185). Type III (one canal dividing and then rejoining) was observed in 6.5% of central incisors, 5.5% of lateral incisors, and 3.6% of canines (Figure 6). Type V (one canal dividing into two) was present in 5.5% of central incisors, 5.0% of lateral incisors, and 2.0% of canines.

**Table 4:** Prevalence and Fan’s classification of C‐shaped canals by tooth type and root level (*n* = 1104).

Tooth type	Total teeth (*n*)	C‐shaped prevalence, *n* (%)	Coronal level^a^	Middle level^a^	Apical level^a^	Clinical implication
Central incisor	200	0 (0.0)	—	—	—	Not observed
Lateral incisor	201	0 (0.0)	—	—	—	Not observed
Canine	196	0 (0.0)	—	—	—	Not observed
First premolar	181	**13 (7.2)**	C1 (62%)	C2 (54%)	C3 (62%)	Highest prevalence: modified instrumentation required
Second premolar	150	5 (3.3)	C1 (60%)	C2 (40%)	C3 (60%)	Moderate prevalence: careful assessment needed
First molar	82	0 (0.0)	—	—	—	Not observed
Second molar	94	**6 (6.4)**	C1 (67%)	C2 (50%)	C3 (50%)	Second highest; complex anatomy
Total	**1104**	**24 (2.2)**	**C1 (62.5%)**	**C2 (50.0%)**	**C3 (58.3%)**	Circumferential filing, ultrasonic irrigation recommended

*Note:* Percentages show most common type at each level among C‐shaped teeth. Pattern shows C‐shaped canals tend to divide apically (C3 most common at apex: 58.3%). The percentages for Fan classifications at the coronal, middle, and apical levels are calculated among the C‐shaped teeth assessed at each respective level. The percentages should not be interpreted as mutually exclusive categories across levels. Bold values indicate the overall totals and summary percentages for the study sample, including the distribution of Fan classification patterns. Bold formatting is used for emphasis only and does not denote statistical significance.

^a^Fan’s classification: C1 = continuous C‐shaped canal; C2 = semicolon‐shaped with separation; C3 = two or three separate canals.

First premolars demonstrated greater morphological variability: Type I 48.1% (*n* = 87), type V 33.7% (*n* = 61), type III 7.2% (*n* = 13), type IV 3.9% (*n* = 7), and Other 7.2% (*n* = 13). Second premolars predominantly exhibited type I configuration (80.7%, *n* = 121), with type V present in 8.0% (*n* = 12), type III in 5.3% (*n* = 8), type IV in 2.7% (*n* = 4), and other in 3.3% (*n* = 5).

Due to the limitations of Vertucci classification in describing multirooted anatomy, 98.8% (*n* = 81) of first molars and 98.9% (*n* = 93) of second molars were classified as “Other,” requiring Ahmed classification for an accurate morphological description.

### 3.4. Ahmed Classification of Canal Configurations

The Ahmed classification system revealed distinct anatomical patterns across tooth types (Table 3). Among all 1104 teeth, the single canal configuration (^1^) was most prevalent, accounting for 61.2% (*n* = 676) of the sample. This was followed by a temporary bifurcation pattern (^121^) in 13.0% (*n* = 143), a single canal in each root of molars (M^1^D^1^) in 8.2% (*n* = 91), and a bifurcation pattern (^12^) in 7.9% (*n* = 87). Additional configurations included M^2^D^1^ in 3.9% (*n* = 43), M^2^D^121^ in 1.4% (*n* = 16), two separate canals (^2^) in 0.6% (*n* = 7), M^1^D^12^ in 0.7% (*n* = 8), and various rare configurations, each representing less than 0.5% of the sample. Tooth‐specific patterns were evident. The ^121^ configuration was predominantly observed in anterior teeth, particularly central incisors (32.5%, *n* = 65) and lateral incisors (26.4%, *n* = 53). The ^12^ bifurcation pattern was most common in first premolars (17.1%, *n* = 31). Among molars, M^1^D^1^ (single canal in each root) was the most frequent configuration, present in 39.0% (*n* = 32) of first molars and 62.8% (*n* = 59) of second molars. The classic molar pattern M^2^D^1^ (two canals in the mesial root and one in the distal root) was observed in 23.2% (*n* = 19) of first molars and 25.5% (*n* = 24) of second molars. Complex configurations such as M^2^D^121^ were identified in 15.9% (*n* = 13) of first molars and 3.2% (*n* = 3) of second molars.

### 3.5. C‐Shaped Canal Prevalence and Distribution

C‐shaped canal configurations were identified in 2.2% (*n* = 24) of all mandibular teeth examined (Table 4). Distribution by tooth type revealed the highest prevalence in first premolars (7.2%, *n* = 13), followed by second molars (6.4%, *n* = 6) and second premolars (3.3%, *n* = 5). No C‐shaped canals were observed in anterior teeth (central incisors, lateral incisors, and canines) or first molars. According to Fan’s classification system, the C‐shaped canal morphology varied by root level. At the coronal level, type C1 (continuous C‐shaped configuration) was most common (62.5%, *n* = 15), followed by type C2 (semicolon‐shaped with separation, 25.0%, *n* = 6) and type C3 (two or three separate canals, 12.5%, *n* = 3). At the middle root level, type C2 predominated (50.0%, *n* = 12), followed by type C1 (33.3%, *n* = 8) and type C3 (16.7%, *n* = 4). At the apical level, type C3 was most prevalent (58.3%, *n* = 14), followed by type C2 (29.2%, *n* = 7) and type C1 (12.5%, *n* = 3), indicating that C‐shaped canals tend to divide into separate canals apically.

### 3.6. Gender‐Based Analysis

Statistical analysis revealed no significant differences between male and female patients in root and canal morphology. Root number distribution showed no gender‐based variation (*p* = 0.67). Root type distribution (separate versus fused) demonstrated no significant difference between genders (*p* = 0.54). Canal configuration patterns according to both Vertucci and Ahmed classifications showed no statistically significant gender differences (*p* = 0.82). C‐shaped canal prevalence was 2.3% in males and 2.1% in females, with no significant difference (*p* = 0.89). Two‐rooted premolar prevalence was 3.8% in males and 3.6% in females, showing no significant gender variation (*p* = 0.91).

## 4. Discussion

This comprehensive CBCT study of 1104 mandibular teeth from 150 Saudi patients provides valuable clinical anatomical data. The large sample size and high‐resolution imaging (0.15 mm voxel size) with rigorous calibration (interobserver *κ* = 0.82–0.84 and intraobserver *κ* = 0.86–0.93) ensure data reliability.

The prevalence of single‐rooted mandibular teeth (83.2%) and two‐rooted teeth (16.6%) is consistent with Middle Eastern populations [4, 17, 18] but differs substantially from East Asian populations where two‐rooted premolars and three‐rooted molars are significantly more common [2, 8, 19]. Al‐Fouzan [17] reported 5.8% two‐rooted premolars in Saudi patients, slightly higher than our 3.3%, while Mashyakhy et al. [18] reported 7.1% in the Jazan region, suggesting regional variation within Saudi Arabia. Reported differences in root and canal morphology across populations should be interpreted cautiously, considering variations in sampling methods, tooth selection, imaging protocols, and demographic characteristics. A literature review of mandibular first premolars highlighted the complexity of their root and canal morphology and the need for careful evaluation before root canal treatment [20].

The extremely low prevalence of three‐rooted and four‐rooted mandibular molars (combined 0.3%) is consistent with other Middle Eastern populations [4, 17] but markedly different from East Asian populations, where prevalence ranges from 2.8% to 12.9% [2, 8, 19]. These differences have practical implications for endodontic education and clinical practice, emphasizing the need for population‐specific anatomical knowledge.

A CBCT study of mandibular first and second molars in a Brazilian population also documented variations in root number, canal configurations, and C‐shaped anatomy [21].

When compared to the conventional Vertucci classification [1], the Ahmed method [6] showed descriptive detail from a categorization standpoint, especially for teeth with many roots. Vertucci’s method is still useful for single‐rooted anatomy, but it is not flexible enough to describe the mesial and distal root configurations separately. Anatomical clarity and clinical communication were improved by the Ahmed system’s alphanumeric format, which allowed for proper reporting of patterns like M^1^D^1^ and M^2^D^1^ as well as more complicated combinations like M^2^D^121^. Implementing this method in practice and research might enhance the uniformity and enable insightful comparisons between studies.

The overall C‐shaped canal prevalence (2.2%) with the highest occurrence in first premolars (7.2%) differs markedly from East Asian populations, where second molar prevalence can exceed 30% [2, 8, 14]. Fan et al. [14] reported 32.7% C‐shaped second molars in Chinese populations, approximately five times higher than our finding. Middle Eastern populations show prevalence rates of 2%–8% [4, 17, 18], consistent with our findings. Al‐Qudah and Awawdeh [4] found 3.2% C‐shaped molars in Jordanians, while Al‐Fouzan [17] reported 4.1% in Saudi patients, suggesting shared genetic influences on root canal morphology.

The 7.2% prevalence of C‐shaped canals in mandibular first premolars has important clinical implications. C‐shaped canals present unique challenges due to complex three‐dimensional anatomy, with continuous ribbon‐like connections making complete debridement and obturation difficult using conventional techniques [14, 22, 23]. Clinicians should maintain heightened awareness of this variation and employ modified instrumentation techniques, including circumferential filing and rotary instruments designed for oval canals [22, 23]. Enhanced irrigation protocols using ultrasonic activation can improve penetration into fins and anastomoses characteristic of a C‐shaped anatomy [23]. Preoperative CBCT imaging should be considered when C‐shaped anatomy is suspected.

Clinical professionals treating patients with characteristics similar to those included in this Saudi clinical sample should anticipate that the majority of their mandibular teeth will be single‐rooted, but they should also be aware of significant differences, especially C‐shaped premolar configurations and intricate canal patterns in molars. To avoid missing canals and insufficient debridement, subtle radiography indicators like sudden canal disappearance or unusual root outlines should raise suspicion of an extra or abnormal architecture. When clinical observations or conventional radiographs indicate complexity, selective preoperative CBCT imaging may be necessary because three‐dimensional evaluation can improve treatment planning and diagnostic precision. Additionally, anatomical accuracy, clinical communication, and case management may all be enhanced by integrating modern methods like the Ahmed categorization into standard documentation.

This study has several limitations that should be acknowledged. First, the retrospective cross‐sectional design and single‐center setting limit the generalizability of the findings. Moreover, because CBCT examinations were obtained only for specific clinical indications at a tertiary referral dental center, the study population represents a selected Saudi clinical sample rather than the general Saudi population, and selection bias cannot be excluded. Second, although the individual tooth was considered the unit of analysis, multiple teeth were evaluated from the same patient, resulting in clustered observations that are not completely independent. The inferential analyses (chi‐square tests) assumed statistical independence and did not account for within‐patient clustering, which may have led to underestimated standard errors and overestimation of statistical significance. Accordingly, the reported *p*‐values should be interpreted with caution and regarded as exploratory rather than confirmatory. Future studies should employ statistical approaches that account for clustered data, such as generalized estimating equations or mixed‐effects models, to provide more robust estimates of associations. Third, although CBCT provides high‐resolution three‐dimensional imaging and is superior to conventional radiography for evaluating root canal morphology, it may still underestimate fine anatomical details, including lateral canals, accessory canals, and apical ramifications.

## 5. Conclusions

This CBCT study demonstrated that mandibular teeth in this Saudi clinical sample exhibited considerable anatomical variation, particularly in premolars and molars. The Ahmed classification system provided a more detailed description of the complex root canal morphology than the Vertucci classification. These findings may assist clinicians in improving diagnosis and treatment planning; however, given the retrospective single‐center design and the use of CBCT examinations obtained for clinical indications, caution is warranted when generalizing the results beyond this clinical sample. Further multicenter, population‐based studies are recommended to validate these findings.

## Author Contributions

**Ahmed A. Almalaq**: conceptualization, methodology, formal analysis, supervision, writing – original draft, writing – review and editing. **Dalal H. Alshammary, Khaled M. Bendary, Firas J. Shaini, Ahmed Alrusyni, Wesam F. Mohamed, Reem A. Alkhamsan, Mahdi M. Al Wusaybie, Khalid N. Alshammari, Turki M. Alazmi, Tahani M. Alharbi, Salem A. Alhamandi, and Faisal A. Alrashid**: investigation, data curation, writing – original draft. **Ahmed A. Madfa**: validation, writing – review and editing.

## Funding

This study did not receive any specific funding.

## Disclosure

All authors have read and approved the final version of the manuscript. Ahmed A. Almalaq had full access to all of the data in this study and takes complete responsibility for the integrity of the data and the accuracy of the data analysis.

## Ethics Statement

Ethical approval was obtained from the Hail Dental Centre, Hail Health Cluster, Ministry of Health, Saudi Arabia (Approval Number 2019‐26). The requirement for informed consent was waived because this retrospective study involved the analysis of previously acquired and anonymized CBCT records. All methods were performed in accordance with the Declaration of Helsinki.

## Conflicts of Interest

The authors declare no conflicts of interest.

## Data Availability

The data that support the findings of this study are available upon request from the corresponding author. The data are not publicly available due to privacy or ethical restrictions.
